# Comparison of Axillary and Tympanic Temperature Measurements in Children Diagnosed with Acute Otitis Media

**DOI:** 10.1155/2016/1729218

**Published:** 2016-08-28

**Authors:** Hatice Hilal Doğan, Rabia Gönül Sezer, Tarık Kırkgöz, Abdulkadir Bozaykut

**Affiliations:** Department of Pediatrics, Zeynep Kamil Maternity and Childrens' Diseases Training and Research Hospital, Istanbul, Turkey

## Abstract

*Background*. Acute otitis media [AOM] may affect the accuracy of tympanic temperature measurements. We aimed to compare tympanic temperature measurements in patients with AOM against control groups, as well as compare the tympanic temperatures with axillary thermometry.* Methods*. This is a prospective, observational study. Patients from pediatric outpatient and emergency clinics who were diagnosed as single-sided AOM were included consecutively in the study. Normal ears of patients and children having the same age and gender who were not diagnosed as AOM were also studied as controls.* Results*. In patients with AOM, infected ears had higher temperatures than normal ears with a mean of 0.48 ± 0.01°C. There was no significant difference between the right and left tympanic temperatures in control group. Compared with axillary temperature, the sensitivity of tympanic temperature in the infected ear was 91.7% and the specificity was 74.8%.* Conclusion*. Comparisons of axillary and tympanic temperatures in children with AOM during the active infection concluded higher tympanic temperatures in infected ears. We suggest that the higher tympanic temperatures, approximately 0.5°C in our study, in infected ears may aid in diagnosis of patients with fever without a source in pediatric clinics.

## 1. Introduction

Acute otitis media (AOM) is a common upper respiratory tract infection in children; 90% of children have at least one attack until two years of age [1]. Most frequent presenting symptoms are fever and otalgia [2, 3]. Children usually present with a history of rapid onset ear pain. However, in young children, otalgia is suggested by crying, high fever, rubbing/holding of the ear, or changes in the child's sleep or behavior pattern [4]. Fever is present in 70% of infants aged under 12 months and in fewer than half of older children [5].

Body temperature measurement is a routine part of clinical assessment in pediatric patients. Tympanic membrane temperature measurement is an easy-to-use, quick, widely acceptable, comfortable, and hygienic method [6]. These advantages made it a first-line application with noncooperative pediatric patients and in pediatric emergency departments. Tympanic thermometer detects infrared energy emitted by the tympanic membrane. Hypothalamus and tympanic membrane share arterial blood supply from carotid artery, so tympanic temperature is believed to reflect core temperature. Studies report tympanic measurement to be as accurate as axillary thermometry [6, 7]. Infrared tympanic thermometer readings were reported to be within the limits of agreement with mercury-in-glass thermometers for axillary recordings [8].

Studies about the accuracy of tympanic temperature measurements in patients with AOM have controversial results [7, 9–13]. Acute otitis media may affect the accuracy of tympanic temperature measurement.

In this study, we aimed to compare tympanic temperature measurements in patients diagnosed as unilateral AOM with their normal ears and the control group and to compare the tympanic temperatures with axillary thermometry.

## 2. Materials and Methods

The study was approved by the local ethics committee (Zeynep Kamil Maternity and Childrens' Diseases Hospital Ethics Committee; name of chairperson: Professor Ayşenur Celayir, MD, date: 19.07.2013; approval number: 72) and informed parental consent was obtained from parents or the legal guardians.

This is a prospective, observational study. Patients from pediatric outpatient and emergency clinics were included from August 2013 to February 2014. Patients aged between 1 month and 18 years who were diagnosed as single-sided AOM were included consecutively in the study. Every patient's gender, age, symptoms, and axillary and tympanic temperatures from both ears were recorded. Patients diagnosed as AOM were also recorded for the infected ear site. We included two control groups: (a) normal ears of patients and (b) children having the same age and gender with AOM patients (there was no one-to-one matching between groups), with any diagnosis other than upper respiratory tract infections, who were not diagnosed as AOM, and who had infections such as urinary tract infections and acute gastroenteritis, either with fever or not.

Acute otitis media is diagnosed based on clinical examination in accordance with published criteria: mild bulging of tympanic membrane with recent onset of earache with a duration of less than 48 hours or intense erythema of tympanic membrane; moderate to severe bulging of the tympanic membrane or newly occurring otorrhea not caused by otitis externa [4]. All the patients were examined for AOM criteria by the same physician (TK) with otoscope (Riester serial number 30041, Ri Scope L: Germany).

Axillary and tympanic temperatures higher than 37.2°C and 38°C were considered as high fever, respectively [14]. Trained children's nursing staff carried out temperature measurements. All nurses were blinded for the study group, either control or AOM patient, and the site of infected ear. Tympanic measurements were taken from both ears according to the manufacturers' recommendations; measurement time is one second. Measurements were taken 3 times and the maximum values were used for calculations to prevent wrong measurements from wrongly placed device in the external auditory canal. Braun IRT 4020 ThermoScan (Braun GmbH, Kronberg, Germany) and Braun Digital Thermometer PRT1000 (Braun GmbH, Kronberg, Germany) were used for tympanic and axillary measurements, respectively. All nursing staff were familiar with the devices and experienced with the manufacturers' instructions.

Exclusion criteria were newborns, presence of bilateral AOM, presence of effusions, parents who do not give consent for the study, presence of wax in the external auditory canal, external otitis, and perforated tympanic membranes.

### 2.1. Statistical Analysis

The analysis was done by SPSS, version 15 [SPSS Inc., Chicago, IL, USA]. Comparisons (a) between axillary and tympanic temperatures and (b) between right and left ears were done using Student* t*-tests and chi-square. Primary outcome was to determine the effect of AOM in both tympanic measurements. Secondary outcomes were comparisons with patients with no AOM and comparison of tympanic measurements with axillary measurements.

## 3. Results

A total of 301 children were included in the study: 151 AOM and 150 controls. The patients' age ranged between 4.5 months and 14 years (mean age = 3.9 ± 3.31 years). Differences between the age, gender, and axillary temperatures of the AOM and control groups were not statistically significant (*p* > 0.05). In the AOM group, the most frequent presenting symptoms were earache, fever, and agitation with percentages of 52.5, 33.7, and 6.9, respectively. Young children, age under 5 years, mostly had fever, whereas children older than 5 years had earache.

Right and left ear temperatures of AOM group, regardless of the infected site, were higher than temperatures of right and left ears of control group (*p* < 0.001). In patients with AOM, infected ears had higher temperatures than normal ears with a mean of 0.48 ± 0.01°C (Table 1). The mean right and left ear tympanic measurements in control group were 36.87 ± 0.59 and 36.92 ± 0.58, respectively. There was no significant difference between the right and left tympanic temperatures in control group (*p* = 0.45).

Compared with axillary temperature, the sensitivity of tympanic temperature in the infected ear was 91.7% and the specificity was 74.8% (Table 2).

## 4. Discussion

Temperature measurement is one of the vital signs in pediatric practice. Tympanic and axillary regions are both acceptable and noninvasive areas. Tympanic membrane is believed to be one of the most reliable regions for detection of core temperature [15]. Its safety is not clear in patients having AOM. In this study, we detected higher tympanic temperatures in infected ears compared to normal ears of the same patients with a mean of 0.48 ± 0.01°C.

Body temperature differs based on time of the day, measurement site, and the technique used; there is no single gold standard technique for measurement. Invasive techniques that can be used in intensive care units would not be suitable for daily clinical use. Many studies search for the answer of the effect of ear pathology on the results of tympanic thermometry [9–13]. The results are hard to compare with different patient groups and different diagnosis. The studies about the accuracy of tympanic temperature measurements in patients with AOM also have conflicting results [7, 9–13]. Some authors reported higher tympanic temperatures in AOM patients (9–11), while others did not report any significant difference (12, 13).

Studies with higher temperatures in infected ear compared to normal ear temperatures in AOM patients, similar to our study, reported a difference range of 0.1–0.6°C [9–11]. A study with 67 children with suppurative AOM had higher temperature with a mean of 0.38°F in the infected ear [9]. The same study did not report any difference in temperatures of 17 children with nonsuppurative AOM [9]. Both the sample size and the different diagnosis may have caused this difference; sample size of 17 is likely not to have been sufficient to demonstrate significance. Also comparisons of tympanic temperatures with rectal or oral temperatures were not significantly different [9]. In 108 patients with unilateral AOM, infected ears had higher tympanic temperatures than uninfected ears with a mean of 0.2 ± 0.9°F (0.1 ± 0.5°C) [10]. However, a similar difference between right and left ears was also noted in patients without AOM. Tympanic temperatures in patients with AOM were not higher than the measurements in patients without AOM; also wax did not significantly affect tympanic temperatures [10]. The reference temperatures were obtained from either oral or rectal route. The sensitivity (45.4%), specificity (17.4%), accuracy (24.3%), positive predictive value (15.1%), and negative predictive value (49.6%) for tympanic temperature in diagnosing AOM were all less than 50% [10]. Chamberlain et al. studied 184 children in pediatric emergency department and elevation of tympanic temperature of approximately 0.1°C was detected within patient comparisons with AOM (*p* < 0.05) [11]. The authors compared tympanic temperatures with oral or rectal temperatures depending on the age of the child. Studies differ in sample size, patients age groups, patients from outpatient or emergency clinics, and different diagnostic criteria for AOM which may all affect study results and within patient comparisons. We suggest that the higher tympanic temperatures, approximately 0.5°C in our study, in infected ears may aid in diagnosis of patients with fever without a source in pediatric clinics.

Many studies searched for the relationship between tympanic and rectal, oral, or axillary temperatures in ear pathologies. Seventy-five children with AOM were compared with 176 noninfected children based on auditory canal and rectal temperatures. Although the temperatures were higher in the otitis group, auditory canal temperatures were not different between infected and uninfected ears [16]. Comparison of tympanic and axillary temperatures in 90 children with exudative otitis media and 20 otologically healthy children revealed higher temperature differences between tympanic and axillary temperatures in infected children than in healthy children [17]. Our study results revealed higher tympanic temperatures in infected ears with a comprehensive control group. Muma et al. evaluated 224 patients in pediatric emergency department; otitis media did not affect comparisons of rectal, axillary, and tympanic membrane measurements [12]. Ninety-five children having otitis media with effusion were evaluated preoperatively by axillary and transtympanic measurement [13]. The authors reported no difference between the body temperatures of transtympanic and axillary route in the presence of effusion [13]. In our study, we included children with infection, acute clinical signs, or fever from the emergency department and outpatient clinics. It is obvious that children with infection or fever will not be operated and elective general anaesthesia will be postponed.

Researchers studied the effect of ear pathologies, from presence of wax in the external auditory canal to even major ear surgery, on tympanic temperatures. Minor ear surgery was not found to affect tympanic temperature [18]. Robb and Shahab and Mandell et al. studied children preoperatively with no infection symptoms or signs [13, 18]. This may be the main reason for the different study results.

García Callejo et al. studied the influence of different otoscopic findings on tympanic temperatures [19]. Study groups included occluded ear with wax, acute otitis externa, nonsuppurative AOM, nonactive central tympanic perforation, transtympanic drain, ears operated with attic antrotomy or radical mastoidectomy, and healthy normal otoscopy. Acute otitis externa increased the tympanic temperature with a mean of 0.36°C; presence of cerumen decreased with a mean of 0.62°C; nonsuppurative otitis media, fluid in ear, tympanic perforation, and ventilation tubes did not affect the results [19]. They studied only nonsuppurative AOM; there were no AOM in the study group, but the authors clearly showed how the tympanic measurements are affected by different otologic pathologies.

As a limitation, infrared thermometers may not measure the exact temperature accurately [20]. There are studies suggesting using tympanic home-use thermometer measurements as screening method but not for decision making [8]. Also, comparison of tympanic measurements with a gold standard measurement for temperature instead of axillary would be more reliable.

Literature supports the relationship between higher tympanic temperatures in children with AOM during the active infection. Whether tympanic temperature is reliable to use in AOM and whether these findings aid in diagnosis of ear infections, or help in diagnosis, especially in young infants who do not have a localizing sign, are questions that remain to be answered.

In this study, the infected ears had an approximately 0.5°C higher temperature than normal ears, and tympanic temperatures had a sensitivity of 91.7% of fever compared to axillary temperatures. We suggest that this difference between measurements can aid in diagnosis of AOM in children.

## Figures and Tables

**Table 1:** Comparison of the temperatures (°C) of infected ear and the normal ear in patients with acute otitis media.

	Mean ± SD	Minimum	Maximum	*p* value
Infected ear	37.86 ± 0.93	35.8	40.1	<0.001
Normal ear	37.38 ± 0.92	35.8	40	

**Table 2:** The associations between axillary and tympanic temperatures in patients with acute otitis media.

		Axillary temperature < 37.2		Axillary temperature ≥ 37.2		Chi-square	*p* value
		*n*	%	*n*	%		
Infected ear temperature	≥38°C	26	25.2	44	91.7	55.09	<0.001
	<38°C	77	74.8	4	8.3		

Normal ear temperature	≥38°C	9	8.7	36	74	68.71	<0.001
	<38°C	94	91.3	12	25		
